# The Impact of Trunk Function and Lower Limb Paralysis on Independence in Activities of Daily Living Among Stroke Patients

**DOI:** 10.7759/cureus.79430

**Published:** 2025-02-21

**Authors:** Takato Nishida, Shun Sawai, Shoya Fujikawa, Ryosuke Yamamoto, Yusuke Shizuka, Takayuki Maru, Kotaro Nakagawa, Hideki Nakano

**Affiliations:** 1 Department of Physical Therapy, Faculty of Rehabilitation and Care, Seijoh University, Tokai, JPN; 2 Graduate School of Health Sciences, Kyoto Tachibana University, Kyoto, JPN; 3 Department of Rehabilitation, Kyoto Kuno Hospital, Kyoto, JPN; 4 Department of Rehabilitation, Tesseikai Neurosurgical Hospital, Shijonawate, JPN; 5 Department of Physical Therapy, Faculty of Health Sciences, Kyoto Tachibana University, Kyoto, JPN; 6 Department of Rehabilitation, Junshinkai Kobe Hospital, Kobe, JPN; 7 Department of Rehabilitation, Nagashima Neurosurgery Rehabilitation Clinic, Osaka, JPN

**Keywords:** activities of daily living, hierarchical regression analysis, lower limb paralysis, stroke, trunk function

## Abstract

Background: Stroke is a major global health issue, and many patients experience motor paralysis and sensory impairments that affect their independence in the activities of daily living (ADLs). Trunk and lower limb functions are crucial in poststroke ADL independence. Although these two functions are closely related, a few studies have evaluated them in combination, and the importance of assessment methods that consider their mutual relationship has not been thoroughly examined. In this study, we aimed to clarify the degree to which trunk function and lower limb paralysis impact ADL independence when evaluated individually versus in combination, through a hierarchical regression analysis, and to verify the significance of the combined assessment of both functions.

Methods: This cross-sectional study included 51 patients with first-ever stroke and hemiplegia. Trunk function was assessed using the Trunk Impairment Scale, lower limb paralysis was evaluated using the Brunnstrom recovery stage for the lower extremities, and ADL independence was measured using the Functional Independence Measure. Hierarchical regression analysis was conducted to examine the impact of trunk and lower limb functions on ADL independence.

Results: Across two regression models, the assessment of trunk and lower limb function in combination significantly improved the accuracy in reflecting ADL independence compared with the assessment of each function individually (p < 0.05).

Conclusions: The combined evaluation method, which assesses both trunk function and lower limb motor paralysis, more accurately reflected ADL independence, suggesting its usefulness as an assessment method in the rehabilitation of patients with stroke.

## Introduction

Stroke, the second most common cause of death and third most common cause of disability, is a leading global health issue [1,2]. Between 1990 and 2019, the incidence and prevalence of stroke increased by 18.5% and 32%, respectively. In 2019 alone, approximately 6.1 million people died from stroke worldwide, highlighting its significant health burden [2]. The particularly high incidence and mortality rates of stroke in low-income regions contribute to health disparities, emphasizing the need for global initiatives in stroke prevention and treatment [3].

Stroke causes a variety of functional impairments, such as motor paralysis, sensory deficits, and speech disorders, which hinder the recovery of independence in activities of daily living (ADLs) and walking ability [4]. Improving ADL independence is a primary goal in poststroke rehabilitation, requiring a multidimensional assessment of functions. In particular, accurate evaluation of trunk function and lower limb paralysis is crucial for developing effective treatment programs [5-8].

Trunk function plays multiple roles, including assisting with support against gravity, respiratory function, and postural control in response to both internal and external disturbances [9]. Impairments in trunk support and postural control significantly affect the recovery of ADL and walking ability in patients with stroke [10,11]. Furthermore, acute-phase trunk function is strongly associated with independence in ADL six months after stroke onset, and the evaluation of trunk function can predict postdischarge balance and mobility abilities [12,13].

Similarly, lower limb function plays a critical role in ADL independence. Lower limb paralysis, weight-bearing on the nonparalyzed side, and lower limb muscle strength have been associated with walking ability and ADL independence in patients with stroke [14,15]. This is particularly evident in ADL tasks that require antigravity activities, such as walking and maintaining a standing posture. Additionally, improvements in upper and lower limb paralysis have been linked to enhanced ADL independence [16,17]. A decline in lower limb function, including motor paralysis, poses a serious challenge to walking and ADL independence in patients with stroke. As such, evaluating lower limb function to facilitate ADL recovery is warranted. Both trunk and lower limb functions have been individually associated with ADL independence. In ADL tasks that require significant standing activity, coordinated movements between the trunk and lower limbs are crucial [18]. Patients with stroke, who often experience motor impairments on one side of the body, may rely more heavily on the interrelationship between trunk and lower limb functions than do healthy individuals.

However, most previous studies have evaluated trunk and lower limb functions separately, and comprehensive assessment methods considering their interrelationship have been insufficiently investigated [18]. Additionally, some studies focusing on the impact of trunk function on ADL independence did not include ADL tasks involving mobility, thereby limiting the practicality of the evaluations [5]. To verify the importance of comprehensive assessment methods that account for the interaction between trunk and lower limb functions in stroke rehabilitation, the incremental addition of functional evaluation items and the use of practical ADL measures are necessary to assess the impact on ADL independence.

Therefore, in this study, we aimed to clarify how trunk function and lower limb paralysis affect ADL independence, both when evaluated individually and in combination, through stepwise analysis. We also sought to verify the importance of a combined assessment method that integrates both functions. We hypothesized that a combined evaluation of trunk and lower limb functions would offer a more accurate ADL independence prediction than separate evaluations of these functions.

This article was previously posted on the medRxiv preprint server on December 3, 2024.

## Materials and methods

Participants

This cross-sectional study included stroke patients who were transferred from an acute care hospital to a specialized rehabilitation hospital for inpatient treatment between July 2017 and October 2018. This study was conducted at Aichi Prefecture Saiseikai Rehabilitation Hospital, a specialized rehabilitation facility. The inclusion criteria were as follows: adult male and female patients with hemiparesis due to a first-ever ischemic or hemorrhagic stroke who were fully conscious at the time of enrollment. The exclusion criteria included subarachnoid hemorrhage, infratentorial lesions, an inability to comprehend verbal instructions, and a lack of consent to participate in the study.

Ethics declarations

This study was conducted in accordance with the principles of the Declaration of Helsinki and was approved by the Research Ethics Committee of Seijoh University (approval number: 2016C0035; approval date: July 12, 2017) and the Aichi Prefecture Saiseikai Rehabilitation Hospital (approval number: 201705; approval date: March 24, 2017). Informed consent was obtained from all the participants in the study.

Measures

We collected data on age, sex, stroke type, paralyzed side, and days since stroke onset from medical records as basic information and administered the Brunnstrom recovery stage for the lower extremities (BRS-LE) to assess motor paralysis, Trunk Impairment Scale (TIS) to assess trunk function, and Functional Independence Measure (FIM) to assess ADL independence. The principal investigator administered the BRS-LE and TIS on the same day. FIM scores were collected from assessments conducted by the responsible physical therapists, occupational therapists, and nurses during the same period.

Brunnstrom recovery stage for the lower extremities

The BRS-LE, proposed by Brunnstrom, evaluates motor paralysis as a qualitative phenomenon based on changes in movement patterns [19]. It comprises assessments of the upper limbs, fingers, and lower limbs, with the degree of separation of associated and synergistic movements rated from Stage I to VI, where VI indicates the mildest form of motor paralysis. This tool was selected owing to its frequent use in Japan as well as its utility as a common language in research, as it served as the basis for developing scales such as the Fugl-Meyer Assessment and Chedoke-McMaster Stroke Assessment [20,21].

Trunk Impairment Scale

The TIS, developed by Verheyden et al., is a clinical assessment tool designed to evaluate motor dysfunction in the trunk [22]. It consists of three subscales: static sitting balance (7 points), which evaluates stability in a seated position; dynamic sitting balance (10 points), which assesses trunk lateral flexion and pelvic elevation movements in a seated position; and coordination (6 points), which evaluates trunk rotation movements. The total score ranges from 0 to 23 points, with higher scores indicating better performance and trunk function. The reliability and validity of this tool have been thoroughly examined [23]. The internal consistency for each subscale and the total score showed Cronbach's α values ranging from 0.65 to 0.85. Reliability measures revealed intraclass correlation coefficient (2,1) values ranging from 0.87 to 0.96 and intraclass correlation coefficient (3,1) values ranging from 0.85 to 0.99. Regarding predictive validity, significant correlations were found with the Barthel Index (r = 0.86) and Trunk Control Test (r = 0.83). Owing to its high reliability and validity, the TIS is considered a valuable tool for assessing trunk function in patients with stroke. Furthermore, as the TIS has been established as a significant predictor of ADL performance, we adopted it as our trunk function assessment measure in this study.

Functional Independence Measure

The FIM measures practical ADL independence [24]. It comprises 13 motor and five cognitive items, each rated on a 7-point scale ranging from 1 (total assistance) to 7 (independence). The FIM is widely used in Japan’s recovery rehabilitation wards and is also employed in calculating medical fees. Herein, we used the total score for the motor items in the analysis.

Statistical analysis

The Shapiro-Wilk test was used to confirm the normality of the data. We used hierarchical regression analysis to analyze the impact of motor paralysis and trunk function on ADL independence and the forced-entry method to examine variables that explained ADL independence. The dependent variable was the FIM score, and the independent variables were age, days since stroke onset, BRS-LE stage, and TIS score. In Step 1, age, days since stroke onset, and TIS score were entered, followed by the BRS-LE stage in Step 2. An alternate analysis was performed to consider the possible influence of variable order by entering age, days since stroke onset, and BRS-LE stage in Step 1, followed by TIS score in Step 2. To illustrate the efficacy of the model in the analysis, multicollinearity was assessed using the variance inflation factor (VIF), and a Durbin-Watson test was conducted. All statistical analyses were conducted using Statistical Package for the Social Sciences Statistics, Version 29 (IBM Corp., Armonk, NY), with the significance level set at 5%.

## Results

Participant characteristics

The analysis included data from 51 participants, whose characteristics are presented in Table 1. A post hoc power analysis using G*Power (Heinrich-Heine-Universität Düsseldorf, Düsseldorf, Germany) revealed a statistical power of 0.80 and an effect size of f² = 0.16, which suggests that the sample size was sufficient to detect differences.

**Table 1:** Participant characteristics Data are shown as mean ± standard deviation or median (interquartile range), as appropriate BRS-LE: Brunnstrom recovery stage for the lower extremities; TIS: Trunk Impairment Scale; FIM: Functional Independence Measure

Characteristics	Value (n = 51)
Age (years)	71.5 ± 13.3
Sex	
Male	29
Female	22
Stroke type	
Ischemic	29
Hemorrhagic	22
Hemiplegic side	
Left	27
Right	24
Days since stroke onset (days)	40 (28-57)
BRS-LE stage	
I	4
II	8
III	2
IV	9
V	6
VI	22
TIS score	17 (7-20)
FIM motor score	54 (21-77)

Hierarchical multiple regression analysis using the TIS score

Table 2 presents the results of the hierarchical multiple regression analysis using the TIS score as an independent variable and the total motor score of the FIM as the dependent variable. The VIFs for all the independent variables were less than 10.0, indicating that multicollinearity was not a concern. The value of the Durbin-Watson test was 1.80, suggesting that the analytical model used in this study is useful. In Step 1, age, days since stroke onset, and TIS score were entered into the model. The adjusted R² of this model was 0.71, suggesting that these variables had a certain explanatory power for the total motor score of the FIM. Among the independent variables, age (β = -0.45, p < 0.01) and TIS score (β = 2.80, p < 0.01) were significant predictors, whereas days since stroke onset (β = -0.05, p = 0.51) did not contribute significantly to the model. In Step 2, the BRS-LE stage was added to the model. The adjusted R² remained at 0.75, indicating a certain explanatory power for the total motor score of the FIM, similar to in Step 1. Among the independent variables, age (β = -0.39, p = 0.01), TIS score (β = 1.24, p = 0.04), and BRS-LE stage (β = 8.11, p < 0.01) were significant predictors, whereas days since stroke onset (β = -0.02, p = 0.73) did not contribute to the model. Moreover, adding BRS-LE significantly increased the change in F-value from Step 1 (ΔF = 8.81, p < 0.01), indicating that including the BRS-LE stage improved the explanatory power of the model. These results suggest that older age, lower trunk function, and lower limb function were associated with lower ADL independence and that adding the BRS-LE stage to the model provided a better explanation of ADL independence than using the TIS score alone.

**Table 2:** Hierarchical multiple regression analysis using the TIS score p value was calculated using hierarchical multiple regression analysis ^*^p < 0.01 β: unstandardized coefficient; SE: standard error; CI: confidence interval; adjusted R2: adjusted coefficient of determination; ΔF: change in F-value from one model to another; TIS: Trunk Impairment Scale; BRS-LE: Brunnstrom recovery stage for the lower extremities

Step and variables	β	SE	95% CI	p value	Adjusted R^2^	ΔF
Step 1						
Age	-0.45	0.16	-0.77 to -0.12	<0.01	0.71	42.45
Days since stroke onset	-0.05	0.07	-0.19 to 0.10	0.51		
TIS score	2.80	0.27	2.26 to 3.34	<0.01		
Step 2						
Age	-0.39	0.15	-0.69 to -0.08	0.01	0.75	8.81^*^
Days since stroke onset	-0.02	0.07	-0.16 to 0.11	0.73		
TIS score	1.24	0.58	0.07 to 2.41	0.04		
BRS-LE stage	8.11	2.73	2.61 to 13.61	<0.01		

Hierarchical multiple regression analysis using the BRS-LE stage

To examine the influence of the order of independent variable input in the hierarchical multiple regression analysis, the analysis was conducted with the order of TIS score and BRS-LE stage reversed. The results are shown in Table 3. The VIFs for all the independent variables were less than 10.0, which indicates that multicollinearity was not a concern. The Durbin-Watson test value was 1.80, which suggests that the analytical model used in this study is useful. In Step 1, age, days since stroke onset, and BRS-LE stage were entered into the model. The adjusted R² was 0.74, suggesting that these variables had a certain explanatory power for the total motor score of the FIM. Among the independent variables, age (β = -0.36, p = 0.03) and BRS-LE stage (β = 13.37, p < 0.01) were significant predictors, whereas days since stroke onset (β = -0.02, p = 0.77) did not contribute significantly to the model. In Step 2, the TIS score was added to the model. The adjusted R² remained at 0.75, indicating a certain explanatory power for the total motor score of the FIM, similar to that in Step 1. Among the independent variables, age (β = -0.39, p = 0.01), BRS-LE stage (β = 8.11, p < 0.01), and TIS score (β = 1.24, p = 0.04) were significant predictors, whereas days since stroke onset (β = -0.02, p = 0.73) did not contribute to the model. The addition of the BRS-LE stage significantly increased the change in F-value from Step 1 (ΔF = 4.55, p < 0.01), suggesting that adding the BRS-LE stage improved the explanatory power of the model. These results were consistent with those obtained when the TIS score was entered first, indicating that the order of independent variable input did not affect the results. Furthermore, these findings demonstrate that combining TIS and BRS-LE evaluations provided a better explanation of ADL independence than evaluating either variable alone.

**Table 3:** Hierarchical multiple regression analysis with inputs from using the BRS-LE stage p value was calculated using hierarchical multiple regression analysis ^*^p < 0.01 β: unstandardized coefficient; SE: standard error; CI: confidence interval; adjusted R2: adjusted coefficient of determination; ΔF: change in F-value from one model to another; TIS: Trunk Impairment Scale; BRS-LE: Brunnstrom recovery stage for the lower extremities

Step and variables	β	SE	95% CI	p value	Adjusted R^2^	ΔF
Step 1						
Age	-0.36	0.16	-0.67 to -0.04	0.03	0.74	47.36
Days since stroke onset	-0.02	0.07	-0.16 to 0.12	0.77		
BRS-LE stage	13.37	1.21	10.93 to 15.81	<0.01		
Step 2						
Age	-0.39	0.15	-0.69 to -0.08	0.01	0.75	4.55^*^
Days since stroke onset	-0.02	0.07	-0.16 to 0.11	0.73		
BRS-LE	8.11	0.50	2.61 to 13.61	<0.01		
TIS score	1.24	0.36	0.07 to 2.41	0.04		

## Discussion

We analyzed the effects of trunk function and lower limb motor paralysis on ADL independence stepwise, clarifying the extent of their impact when assessed individually and in combination. We also verified the importance of evaluating both functions in combination. The accuracy of reflecting ADL independence was improved when trunk function and lower limb motor paralysis were evaluated together rather than individually. This result suggests that it is important to evaluate the combination of trunk and lower limb functions to rehabilitate patients with stroke.

The study revealed an increase in the change in F-value when both functions were added to the regression model vs. when either trunk function or lower limb motor paralysis was used individually; thus, the combination of functions improved the explanatory power of the model. This may be because the ADLs of patients with stroke involve a variety of movements performed in sitting and standing positions. The FIM includes items such as toileting, transfers, mobility, and stair climbing, all of which require balance and mobility in a standing position [24]. While trunk function is crucial for sitting balance, the lower limb function, particularly the strength of the muscles around the hip joint, is important for standing balance and walking [25,26]. Therefore, practical ADL requires both trunk and lower limb functions. This suggests that combining the evaluation of trunk function and lower limb motor paralysis better explains ADL independence than individual evaluations.

Furthermore, both the lateral and medial motor systems play essential roles in the acquisition of ADL movements after stroke [27]. The lateral motor system mainly controls the distal muscles of the limbs via the corticospinal tract. In contrast, the medial motor system controls the trunk and proximal muscles of the limbs via the corticoreticulospinal and corticorubral tracts. These neural pathways are closely situated, rendering them highly susceptible to simultaneous damage during stroke [28]. From the perspective of the neural fibers involved, this suggests the need to evaluate both trunk and lower limb functions comprehensively.

The relationship between trunk and lower limb functions has been reported in previous studies. Verheyden et al. have shown that trunk stability is a prerequisite for the movement of the head and limbs and that these functions are related to ADL [29]. Hsieh et al. have also investigated the impact of walking ability after stroke on ADL, reporting that the recovery of lower limb function is directly linked to improvements in ADL independence [5]. Based on the results of the present study, it was found that in poststroke rehabilitation, a combined evaluation of both trunk and lower limb function better reflected ADL independence than did evaluating these functions individually. Therefore, to understand the pathophysiology of patients with stroke in clinical practice, it is necessary to evaluate lower limb and trunk function simultaneously rather than independently and plan training.

In previous studies, reports indicated patients in the early acute phase of disease onset [27]. Additionally, there are findings that highlight a decline in lower limb and trunk function, which is involved in the acquisition of ADL at stages beyond the acute phase of the disease [5,30]. However, these studies utilized the Barthel Index to evaluate ADL, and one issue is that practical ADLs, including mobility, have not been adequately assessed. In the present study, hierarchical multiple regression analysis was conducted on the data of patients recovering from stroke, using the FIM score, which reflects practical ADL independence as the dependent variable, and trunk function and lower limb motor paralysis as independent variables. As a result, new findings were obtained regarding the influence of these functions on ADL independence.

One limitation of this study is that the severity of paralysis varied among participants, and differences in paralysis severity may have influenced the results. Future studies should include larger sample sizes and conduct subgroup analyses based on paralysis severity for a more detailed examination. Additionally, major disease-related variables, such as overall disease severity and comorbid medications, were not considered in this study. These factors may have influenced ADL independence and should be considered in future research to provide a more comprehensive understanding of the relationships between trunk function, lower limb function, and ADL performance in stroke patients. Furthermore, while the TIS consists of multiple functional components, this study did not analyze these subcomponents individually. A more detailed investigation into how each element of the TIS, such as static and dynamic sitting balance and coordination, relates to ADL independence may provide further insights. Future studies should consider examining these subcomponents separately to better clarify their respective contributions to functional recovery. In addition, the relatively small sample size (n = 51) may have affected the generalizability of the findings. Although the statistical power was deemed sufficient, future studies should include larger cohorts to enhance the robustness of the results and confirm the findings in broader populations. Finally, the cross-sectional design precluded the evaluation of long-term rehabilitation outcomes. Longitudinal studies are necessary to ascertain the relationship between trunk and lower extremity function and improvements in ADL over time. The clinical assessment tools used in this study were also limited. A more comprehensive evaluation of ADL independence may require an analysis that considers not only trunk and lower extremity function but also upper extremity paralysis, cognitive function, muscle strength, and sensory function. Future studies should integrate these additional factors to enhance the accuracy and applicability of the findings.

## Conclusions

The combined evaluation method, which measures both trunk function and lower limb motor paralysis, more accurately reflected ADL independence, which suggests its usefulness as an assessment method. Future studies should include larger sample sizes, incorporate a longitudinal design, and adopt more multifaceted evaluation methods to examine the effects of trunk and lower limb functions on ADL from multiple perspectives and address the limitations of our study.
